# Graded Associations of Blood Lead and Urinary Cadmium Concentrations with Oxidative-Stress–Related Markers in the U.S. Population: Results from the Third National Health and Nutrition Examination Survey

**DOI:** 10.1289/ehp.8518

**Published:** 2005-11-15

**Authors:** Duk-Hee Lee, Ji-Sun Lim, Kyungeun Song, Yongchool Boo, David R. Jacobs

**Affiliations:** 1 Department of Preventive Medicine and Health Promotion Research Center,; 2 Department of Clinical Pathology, School of Medicine, and; 3 Department of Molecular Biology, School of Medicine, Kyungpook National University, Daegu, South Korea; 4 Division of Epidemiology, School of Public Health, University of Minnesota, Minneapolis, Minnesota, USA; 5 Department of Nutrition, University of Oslo, Oslo, Norway

**Keywords:** cadmium, carotenoid, γ-glutamyltransferase, lead, oxidative stress, vitamin C, vitamin E

## Abstract

Although oxidative stress has been proposed as a mechanism of lead and cadmium toxicity mostly based on *in vitro* experiments or animal studies, it is uncertain whether this mechanism is relevant in the pathogenesis of lead- or cadmium-related diseases in the general population with low environmental exposure to lead and cadmium. We examined associations of blood lead and urinary cadmium levels with oxidative stress markers of serum γ-glutamyltransferase (GGT), vitamin C, carotenoids, and vitamin E among 10,098 adult participants in the third U.S. National Health and Nutrition Examination Survey. After adjusting for race, sex, and age (plus serum total cholesterol in the case of serum carotenoids and vitamin E), blood lead and urinary cadmium levels both showed graded associations, positive with serum GGT and inverse with serum vitamin C, carotenoids, and vitamin E (*p* for trend < 0.01, respectively). These associations were consistently observed among most subgroups: non-Hispanic white, non-Hispanic black, men, women, all age groups, non-drinkers, drinkers, nonsmokers, ex-smokers, current smokers, and body mass index (< 25, 25–29.9, and ≥30). The strong association of blood lead and urinary cadmium levels with oxidative stress markers in this population suggests that oxidative stress should be considered in the pathogenesis of lead- and cadmium-related diseases even among people with low environmental exposure to lead and cadmium.

Lead and cadmium are ubiquitous environmental toxicants that are related to a broad range of physiologic, biochemical, and behavioral dysfunctions (Goyer 1990; Satarug and Moore 2004). Lead and cadmium potentially induce oxidative stress; evidence is accumulating to support the role of oxidative stress in the pathophysiology of lead or cadmium poisoning (Ercal et al. 2001; Stohs and Bagchi 1995). Until now, evidence on lead- or cadmium-induced oxidative stress has been based mostly on *in vitro* experiments (Ding et al. 2000; Gaubin et al. 2000; Kim et al. 2005; Loikkanen et al. 1998) or animal studies (Cabell et al. 2004; Farmand et al. 2005; Fowler et al. 2004; Tandon et al. 2003). Several epidemiologic studies among workers with high occupational exposure to lead have reported associations between lead exposure and oxidative stress markers (Costa et al. 1997; Gurer-Orhan et al. 2004; Kasperczyk et al. 2004).

Concern about environmental exposure to lead or cadmium as a significant public health problem has increased as epidemiologic and experimental evidence has mounted regarding adverse health effects at successively lower levels of lead or cadmium exposure (Davis et al. 1993; Satarug et al. 2003). Recent epidemiologic studies have reported that environmental exposure to lead or cadmium concentration has a graded association with several disease outcomes such as hypertension, peripheral artery diseases, kidney diseases, and cognitive impairment (Elliott et al. 2000; Jarup et al. 1998; Koller et al. 2004; Muntner et al. 2003; Nash et al. 2003; Navas-Acien et al. 2004). Although all these diseases include components of oxidative stress (Hogg 1998), the relevance of oxidative stress to lead- and cadmium-related diseases in the general population with low environmental exposure has been criticized because mechanistic studies are typically conducted at higher doses than the concentrations observed in general population (Navas-Acien et al. 2004).

To explore the possibility that oxidative stress may be involved in the pathogenesis of lead- or cadmium-related diseases in the general population, we examined the cross-sectional associations of blood lead and urinary cadmium levels with serum γ-glutamyltransferase (GGT), vitamin C, carotenoids, and vitamin E in a representative sample of the U.S. population using the Third National Health and Nutrition Examination Survey (NHANES III). We expected a positive association of blood lead or urinary cadmium levels with serum GGT and inverse associations with vitamin C, carotenoids, and vitamin E. Although an abnormal level of serum GGT, a well-known enzyme, is a marker of liver dysfunction or alcohol consumption, serum GGT within its normal range has been recently proposed as an early and sensitive biomarker of oxidative stress (Lee et al. 2004). Finding a threshold above which blood lead and cadmium are associated with oxidative stress in the general population could be helpful in setting safety levels for environmental exposure to these metals.

## Materials and Methods

NHANES III is a national examination study conducted in the United States from 1988 through 1994 by the National Center for Health Statistics of the Centers for Disease Control and Prevention (CDC). It used complex, multistage, stratified, clustered samples of civilian, noninstitutionalized populations 2 months of age and older. A detailed description of survey methods and data collection procedures has been published elsewhere (National Center for Health Statistics 1996).

### Study sample.

Of 18,825 sampled persons ≥20 years of age, 16,573 (88%) attended an examination at a mobile examination center. Pregnant women (*n* = 288) and participants with missing data on serum lead, GGT, vitamin C, carotenoids, vitamin E, or urinary cadmium or creatinine concentrations (*n* = 5,310) were excluded. We additionally excluded those with missing data on confounders (*n* = 1,679). Finally, 10,098 study participants remained for analysis.

### Measurements.

The NHANES III data collection included a standardized home interview followed by a detailed physical examination in a mobile examination center or the participant’s home. Information on a wide variety of sociodemographic, medical history, nutritional history, and family history questions, such as self-reported age, race/ethnicity, sex, history of smoking, alcohol consumption, use of vitamin supplements, and 24-hr dietary recall, were obtained during the home interview.

Venous blood and urine samples were collected and shipped weekly at −20°C. Blood lead levels and urinary cadmium were determined using graphite furnace atomic absorption spectrophotometry. Serum GGT concentration was assayed with a Hitachi 737 Analyzer (Boehringer-Mannheim Diagnostics, Indianapolis, IN, USA) at White Sands Research Center, Alamogordo, New Mexico (USA). Serum extract was prepared in the vial for vitamin C analysis by diluting 500 μL serum with 2.0 mL freshly prepared 6 mg/dL metaphosphoric acid diluent and thoroughly mixing the resulting solution of clear liquid and white precipitated proteins. Serum vitamin C was measured by isocratic high-performance liquid chromatography (HPLC) with electrochemical detection at 650 mV. Serum carotenoids (α-carotene, β-carotene, β-cryptoxanthin, lutein/zeaxanthin, and lycopene) and vitamin E concentrations were measured by isocratic HPLC with detection at 300, 325, and 450 nm using a Waters HPLC system (Waters Chromatography Division of Millipore Corporation, Marlboro, MA, USA) at the NHANES Laboratory. Serum total cholesterol was measured using a Hitachi 704 Analyzer (Boehringer-Mannheim Diagnostics) at the Johns Hopkins University Hospital Lipoprotein Analytical Laboratory (Baltimore, MD, USA) and White Sands Research Center.

### Statistical analysis.

We classified both blood lead and urinary cadmium levels into deciles: Cutoff points of blood lead deciles were 1.0, 1.5, 1.9, 2.4, 2.9, 3.5, 4.2, 5.2, and 7.1 μg/dL and those of urinary cadmium were 0.11, 0.18, 0.25, 0.33, 0.41, 0.52, 0.67, 0.88, and 1.24 μg/g creatinine. Serum GGT and some serum antioxidant vitamin levels were right-skewed, so results are presented as geometric means across deciles of blood lead or urinary cadmium levels. We also present the associations of serum alanine aminotransferase (ALT), a liver-specific enzyme, with blood lead and urinary cadmium levels to compare with those of GGT. We adjusted for potential confounding by linear regression. In this study, the internal validity was a more important issue than was generalization to the total U.S. population; therefore, we did not use a specific analytic method to take into account the sampling frame of NHANES III. When we repeated the analyses considering the sampling frame, the results of weighted results were similar to those of unweighted results. Adjusting variables were race/ethnicity, sex, age (years), education (years), and poverty income ratio. The values of carotenoids and vitamin E were additionally adjusted for cholesterol concentrations because the distribution of these lipophilic compounds across various fat depots in the body is influenced by circulating lipoprotein concentrations. In fully adjusted models, we additionally adjusted for body mass index (BMI; kilograms per square meter), smoking status (nonsmoker, ex-smoker, and current smoker), smoking amount (packs), and alcohol intake (grams per day). We repeated the same analyses after stratifying by race (non-Hispanic white, non-Hispanic black, others), sex (men, women), alcohol consumption status (nondrinker, drinker), smoking status (never smoker, ex-smoker, current smoker), or BMI (< 25, 25–29.9, ≥30 kg/m^2^).

## Results

**Figure 1:**
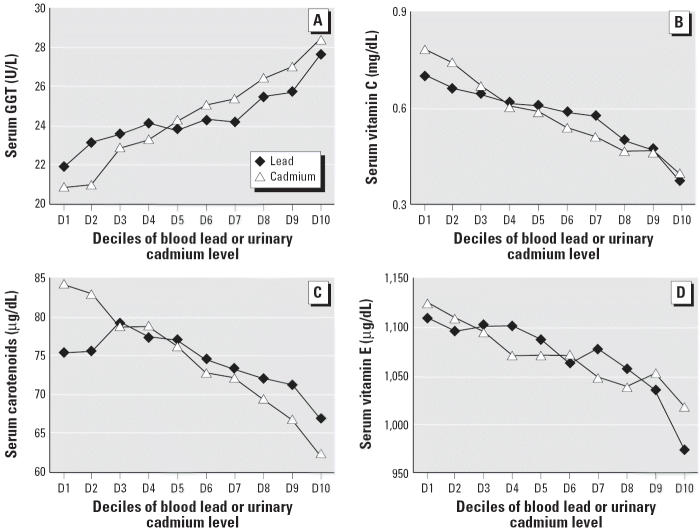
Geometric means of serum GGT (*A*), vitamin C (*B*), carotenoids (*C*), and vitamin E (*D*) by deciles of blood lead or urinary cadmium levels, adjusted for race, sex, age, education, and poverty income ratio. Concentrations of carotenoids and vitamin E were additionally adjusted for serum cholesterol. Serum carotenoids were the sum of α-carotene, β-carotene, β-cryptoxanthin, zeaxanthin/lutein, and lycopene.

Geometric means of blood lead and urinary cadmium among the study subjects were 2.8 mg/dL and 0.37 μg/g creatinine, respectively. The correlation of blood lead and urinary cadmium was *r* = 0.24. After adjusting for race, sex, age, education, and poverty income ratio (plus serum total cholesterol in the case of serum carotenoids and vitamin E), in general, both blood lead and urinary cadmium levels were positively associated with serum GGT, whereas they were inversely associated with serum vitamin C, carotenoids, and vitamin E (*p* for trend < 0.01, respectively) (Table 1, Figure 1). These associations were observed across most deciles of blood lead and urinary cadmium. A partial exception was serum carotenoids and lead, where the association was flat across deciles 1–5; alternatively, a monotonic decrease started at decile 5. When we separately analyzed five carotenoids (α-carotene, β-carotene, β-cryptoxanthin, lutein/zeaxanthin, lycopene), all carotenoids except lutein/zeaxanthin (no association with lead) showed similar trends (data not shown). In contrast, serum ALT, a liver-specific enzyme, was not positively associated with blood lead irrespective of adjustment for serum GGT or with urinary cadmium after adjustment for serum GGT. The positive associations of both blood lead and urinary cadmium with serum GGT were not changed even after adjustment for serum ALT. All these associations were not materially different after additional adjustment for cigarette smoking, alcohol intake, and BMI (data not shown). Additional adjustments for dietary intakes of vitamin C, carotenoids, and vitamin E and supplement intake did not substantially change the results (data not shown). We checked the interactions between blood lead and urinary cadmium on the levels of oxidative stress markers; however, there was no reportable result (data not shown).

When we stratified the associations of blood lead and urinary cadmium levels by serum GGT or vitamin C by race, sex, age, smoking status, drinking status, or BMI, the positive or inverse associations of each with serum GGT levels and vitamin C were similarly observed among most subgroups: non-Hispanic white, non-Hispanic black, men, women, all age groups, nondrinkers, drinkers, nonsmokers, ex-smokers, current smokers, BMI < 25, BMI 25–29.9, and BMI ≥30 kg/m^2^ (Tables 2 and 3). Some *p*-values for interactions were significant, but all β-coefficients were in the same direction, and there was no clear pattern of which subgroups had shallower or steeper slopes. Results of stratified analyses of serum carotenoids or vitamin E were similar to those of vitamin C (data not shown).

## Discussion

In this sample of the U.S. population, we documented that blood lead and urinary cadmium levels across all deciles were positively associated with serum GGT levels, whereas they were inversely associated with serum vitamin C, carotenoids, and vitamin E. These associations were consistently demonstrated in all subgroups. Importantly, the observed increase of serum GGT and decrease of serum vitamin C, carotenoids, and vitamin E occurred at lead or cadmium levels much lower than current safety levels used by environmental and occupational regulatory agencies. For instance, only three study participants had lead levels > 40 μg/dL, the Occupational Safety and Health Administration (OSHA) safety standard for lead in whole blood (OSHA 2003), and only 449 (4.1%) had lead levels > 10 μg/dL, the CDC criterion for elevated blood levels in children and pregnant women (CDC 2002). Similarly, only 22 (0.2%) had urinary cadmium levels > 5 μg/g creatinine, the World Health Organization (WHO) standard for urinary cadmium (WHO 1999).

Lead or cadmium causes oxidative stress by inducing the generation of reactive oxygen species, reducing the antioxidant defense systems of cells via depleting glutathione, inhibiting sulfhydryl-dependent enzymes, interfering with some essential metals needed for anti-oxidant enzyme activities, and/or increasing susceptibility of cells to oxidative attack by altering the membrane integrity and fatty acid composition (Cabell et al. 2004; Ding et al. 2000; Farmand et al. 2005; Fowler et al. 2004; Gaubin et al. 2000; Kim et al. 2005; Loikkanen et al. 1998; Tandon et al. 2003). Consequently, it is plausible that impaired oxidant/antioxidant balance can be partially responsible for the toxic effects of lead or cadmium. Several epidemiologic studies among workers with high occupational exposure to lead have reported positive associations between blood lead levels and oxidative stress markers (Costa et al. 1997; Gurer-Orhan et al. 2004; Kasperczyk et al. 2004).

Despite the biologic plausibility, the relevance of these mechanisms to the general population with low environmental exposure to lead or cadmium has been criticized because mechanistic studies are typically conducted at higher doses than the concentrations observed in the present study (Navas-Acien et al. 2004). However, the present study strongly suggests that oxidative stress is occurring even at low levels of exposure to lead and cadmium. Consequently, it should be considered as a relevant mechanism in the pathophysiology of lead- or cadmium-related diseases, even in those with low environmental exposure to lead or cadmium.

The general population can be exposed to lead in ambient air near industrial and combustion sources, in certain foods, through smoking, and sometimes in drinking water [Agency for Toxic Substances and Disease Registry (ATSDR) 1999b]. Lead exposure has declined substantially in the last two decades since the ban on leaded gasoline (Pirkle et al. 1994). Since NHANES II, the blood lead concentrations declined dramatically in the U.S. population (Pirkle et al. 1994). For example, among the U.S. population 1–74 years of age, mean blood lead concentrations dropped by 78% from 12.8 μg/dL during NHANES II (1976–1980) to 2.8 μg/dL during the first phase of NHANES III (1988–1991). Exposure to cadmium in the general population results from exposure to cigarette smoke, inhalation of ambient air near coal-fired power plants and municipal waste incinerators, and consumption of some foods (highest levels in shellfish, liver, and kidney meats) (ATSDR 1999a).

Serum GGT levels within normal range has been recently proposed as an early and sensitive marker of oxidative stress based on both experimental and epidemiologic studies (Lee et al. 2004). Even though serum GGT was highly correlated with serum ALT in these data (correlation = 0.54), the associations of serum ALT within its normal range with serum antioxidant vitamin levels or C-reactive protein were very different from those of GGT within its normal range. For example, serum GGT was clearly and inversely associated with serum antioxidant vitamins, whereas serum ALT was not associated or sometimes positively associated with them (Lim et al. 2004). In addition, serum GGT was clearly and positively associated with serum C-reactive protein, whereas serum ALT was not positively, if anything inversely, associated with serum C-reactive protein (Lee and Jacobs 2005). Thus, we assert that serum GGT within normal range, different from serum ALT, may be a marker related to oxidative stress.

The strengths of our study include the rigorous methodology and extensive quality control of NHANES procedures, the use of a nationally representative sample, and the consistent findings in most subgroups. Our findings, however, must be interpreted in the context of certain limitations. First, serum GGT and antioxidant vitamins as oxidative-stress–related markers were selected based on availability in NHANES III. In fact, there are various biologic markers that are used to assess oxidative stress that include F_2_ isoprostanes, 8-hydroxydeoxyguanosine, and protein carbonyls (Mayne 2003). These biomarkers give a view of oxidative damage to, respectively, lipid, DNA, and protein but individually do not give a global view of whole-body stress (Mayne 2003). At present, it is unclear which oxidative-stress–related biomarker may be most relevant in oxidative stress induced by lead or cadmium. Second, even though we focused on lead and cadmium in this study, there may be concomitant exposure to other metals that can also induce oxidative stress. However, such coexposure may be more common for workers exposed to high levels of lead or cadmium in occupational settings than to exposure in the general population. Third, our analyses were based on single blood or urine measurements of lead and cadmium, which are imperfect biomarkers of chronic exposure. Environmental exposures, however, are likely to be less changeable than occupational exposures, and single blood or urine levels are frequently used as biomarkers in population studies. Finally, NHANES used spot urine to measure urinary cadmium, not 24-hr urine collection. Even though we adjusted for urinary creatinine to account for urine dilution, the adequacy of correcting for creatinine has been questioned (Ikeda et al. 2003). In our data, the findings using models with and without adjustment for creatinine were similar and do not affect the conclusions.

In conclusion, this study finds evidence of oxidative stress at levels of blood lead and urinary cadmium substantially lower than those labeled as dangerous by OSHA, CDC, or WHO. At present, it is unclear whether slight increases of oxidative stress markers or slight decreases of serum antioxidants increase the future risk of clinical outcomes; nevertheless, this increased oxidative stress might be harmful. Even though it needs confirmation in further studies, our present study suggests that the consumption of antioxidant-rich foods such as fruits or vegetables may be helpful to prevent harmful effects of environmental lead or cadmium exposures. These findings should be borne in mind in thinking about possible adverse effects of lead or cadmium and in setting alert levels for blood lead or cadmium.

## Figures and Tables

**Table 1:** Adjusted^a^ geometric means of serum GGT, vitamin C, carotenoids, and vitamin E by deciles of blood lead or urinary cadmium levels.

	Decile											
	D1	D2	D3	D4	D5	D6	D7	D8	D9	D10	β-Coefficient^b^	*p*-Value for trend
Blood lead levels (μg/dL)	0.7–1.0	1.1–1.5	1.6–1.9	2.0–2.4	2.5–2.9	3.0–3.5	3.6–4.2	4.3–5.2	5.3–7.1	7.2–56		
GGT (U/L)	21.9	23.2	23.6	24.1	23.8	24.3	24.2	25.4	25.8	27.6	0.057	< 0.01
ALT (U/L)	14.5	14.9	15.7	15.5	15.4	14.8	14.9	14.8	14.3	13.9	−0.025	< 0.01
Vitamin C (mg/dL)	0.70	0.66	0.64	0.61	0.61	0.59	0.58	0.50	0.47	0.37	−0.175	< 0.01
Carotenoids (μg/dL)^c^	75	76	79	77	77	75	73	72	71	67	−0.040	< 0.01
Vitamin E (μg/dL)	1,110	1,096	1,100	1,101	1,087	1,062	1,077	1,057	1,035	974	−0.035	< 0.01
Urinary cadmium levels (μg/g creatinine)	0.002–0.11	0.12–0.18	0.19–0.25	0.26–0.33	0.34–0.41	0.42–0.52	0.53–0.67	0.68–0.88	0.89–1.24	1.25–23.4		
GGT (U/L)	20.9	21.0	23.0	23.3	24.2	25.1	25.3	26.4	27.1	28.4	0.053	< 0.01
ALT (U/L)	14.0	14.1	14.9	14.5	15.0	15.0	15.2	15.2	15.4	15.3	0.021	< 0.01
Vitamin C (mg/dL)	0.78	0.74	0.66	0.60	0.59	0.54	0.51	0.47	0.46	0.40	−0.125	< 0.01
Carotenoids (μg/dL)^c^	84	83	79	79	76	73	72	70	67	62	−0.058	< 0.01
Vitamin E (μg/dL)	1,126	1,109	1,094	1,071	1,072	1,071	1,048	1,037	1,053	1,019	−0.013	< 0.01

a Adjusted for race, sex, age, education, and poverty income ratio; concentrations of carotenoids and vitamin E were additionally adjusted for serum cholesterol.

b β-Coefficients: adjusted slopes of the independent variable blood lead or urinary cadmium level (per SD).

c Serum carotenoids were the sum of α-carotene, β-carotene, β-cryptoxanthin, zeaxanthin/lutein, and lycopene.

**Table 2:** Adjusted^a^ slopes of blood lead level (per SD of lead) on serum GGT and vitamin C, stratified by race, sex, age, smoking status, drinking status, or BMI.

		GGT		Vitamin C	
	Sample (*n*)	β-Coefficient	*p*-Value for trend	β-Coefficient	*p*-Value for trend
Race
Non-Hispanic white	4,485	0.031	0.01	−0.183	< 0.01
Non-Hispanic black	2,820	0.078	< 0.01	−0.185	< 0.01
Others	2,793	0.010	0.53	−0.083	< 0.01
*p*-Value for interaction		0.88		< 0.01	
Sex
Men	4,799	0.063	< 0.01	−0.167	< 0.01
Women	5,299	0.049	< 0.01	−0.196	< 0.01
*p*-Value for interaction		0.04		0.76	
Age (years)
20–39	4,178	0.052	< 0.01	−0.128	< 0.01
40–59	2,831	0.068	< 0.01	−0.217	< 0.01
≥60	3,089	0.023	0.03	−0.165	< 0.01
*p*-Value for interaction		< 0.01		0.01	
Smoking
Never	5,016	0.028	0.03	−0.075	< 0.01
Ex	2,550	0.043	< 0.01	−0.161	< 0.01
Current	2,532	0.063	< 0.01	−0.150	< 0.01
*p*-Value for interaction		0.51		< 0.01	
Drinking
No	7,707	0.021	0.02	−0.159	< 0.01
Yes	2,391	0.105	< 0.01	−0.198	< 0.01
*p*-Value for interaction		< 0.01		0.02	
BMI
< 25	3,900	0.093	< 0.01	−0.211	< 0.01
25–29.9	3,567	0.052	< 0.01	−0.141	< 0.01
≥30	2,631	0.034	0.04	−0.172	< 0.01
*p*-Value for interaction		< 0.01		< 0.01	

a Adjusted for race, sex, age, education, and poverty income ratio.

**Table 3:** Adjusted^a^ slopes of urinary cadmium (per SD of cadmium) on serum GGT and vitamin C, stratified by race, sex, age, smoking status, drinking status, or BMI.

		GGT		Vitamin C	
	Sample (*n*)	β-Coefficient	*p*-Value for trend	β-Coefficient	*p*-Value for trend
Race
Non-Hispanic white	4,485	0.073	< 0.01	−0.147	< 0.01
Non-Hispanic black	2,820	0.098	< 0.01	−0.153	< 0.01
Others	2,793	0.009	0.43	−0.082	< 0.01
*p*-Value for interaction		< 0.01		0.70	
Sex
Men	4,799	0.058	< 0.01	−0.140	< 0.01
Women	5,299	0.046	< 0.01	−0.118	< 0.01
*p*-Value for interaction		0.03		< 0.01	
Age (years)
20–39	4,178	0.097	< 0.01	−0.223	< 0.01
40–59	2,831	0.048	< 0.01	−0.145	< 0.01
≥60	3,089	0.014	0.10	−0.081	< 0.01
*p*-Value for interaction		< 0.01		< 0.01	
Smoking
Never	5,016	0.029	0.01	−0.048	< 0.01
Ex	2,550	0.051	< 0.01	−0.069	< 0.01
Current	2,532	0.056	< 0.01	−0.103	< 0.01
*p*-Value for interaction		0.62		< 0.01	
Drinking
No	7,707	0.036	< 0.01	−0.102	< 0.01
Yes	2,391	0.133	< 0.01	−0.247	< 0.01
*p*-Value for interaction		< 0.01		< 0.01	
BMI
< 25	3,900	0.105	< 0.01	−0.188	< 0.01
25–29.9	3,567	0.045	< 0.01	−0.090	< 0.01
≥30	2,631	0.032	0.02	−0.107	< 0.01
*p*-Value for interaction		< 0.01		< 0.01	

a Adjusted for race, sex, age, education, and poverty income ratio.
